# Canine cutaneous and renal glomerular vasculopathy in the Republic of Ireland: a description of three cases

**DOI:** 10.1186/s13620-019-0151-7

**Published:** 2019-11-16

**Authors:** Aimee Hope, Carlos Martinez, Joseph P. Cassidy, Barbara Gallagher, Carmel T. Mooney

**Affiliations:** 1 University College Dublin Veterinary Hospital, University College Dublin, Belfield Campus, Stillorgan Road, Dublin, Ireland; 2 Axiom Veterinary Laboratories, The Manor House, Brunel Road, Newton Abbot, Devon, TQ12 4PB England

**Keywords:** Alabama rot, Skin lesions with azotaemia, Thrombotic microangiopathy

## Abstract

**Background:**

Cutaneous and renal glomerular vasculopathy (CRGV) is a condition of unknown aetiology involving microvascular thrombosis. It has recently been described in over 160 dogs in the United Kingdom and usually has a grave prognosis. To date, this condition has not been described in dogs residing in the Republic of Ireland.

**Case presentation:**

Three dogs presented to University College Dublin Veterinary Hospital (UCDVH) for investigation of rapidly progressive skin lesions. All dogs were diagnosed with CRGV on post-mortem examination. All three dogs had azotaemia on presentation or rapidly developed azotaemia, and all were euthanased because of progression of clinical signs and likelihood of CRGV. One dog was affected by seizure-like episodes and had thrombotic microangiopathy evident within the cerebrum.

**Conclusions:**

CRGV occurs in dogs residing in the Republic of Ireland and is a differential for cases presenting with skin lesions and azotaemia. The histopathological lesions of CRGV can also affect the brain leading to neurological signs such as seizures. Owners and veterinarians should be aware that this condition can occur in dogs in Ireland.

## Background

Cutaneous and renal glomerular vasculopathy (CRGV) is a vascular disease of unknown aetiology associated with cutaneous lesions and acute kidney injury (AKI). The condition was first described in greyhound dogs residing in North America in 1988 [1]. Due to the large numbers of affected greyhounds confined to a specific racetrack in Alabama, the condition was referred to as ‘Alabama Rot’. Later, cases were also reported from other areas in North America [2]. The first reports in Europe were in 2000 and 2002, respectively, concerning a greyhound in the United Kingdom (UK) [3] and a great Dane in Germany [4]. Since that time, details of 30 confirmed cases in the UK evaluated between November 2012 and March 2014 have been reported [5], although this number now exceeds 160 [6]. Cases in the Republic of Ireland have not been published to date.

Clinically, affected dogs present with erosive/ulcerative skin lesions affecting the distal limbs, ventrum, oral cavity and muzzle. Although some dogs may fully recover, typically there is rapid (within days) progression to AKI often with oliguria or anuria [5]. Additionally, common clinicopathological abnormalities include icterus (jaundice), anaemia and thrombocytopenia, although an array of other abnormalities have been documented [1, 2, 4, 5]. Treatment is largely empirical and the prognosis guarded to poor depending on the extent of the AKI [6]. Plasma exchange therapy has been suggested to improve prognosis with two of six cases undergoing such treatment surviving [7].

The histopathological hallmark of CRGV is thrombotic microangiopathy (TMA), most commonly observed in renal glomeruli. This process is characterised by the development of microvascular thrombosis associated with endothelial damage, resulting in consumptive thrombocytopenia, haemolytic anaemia and multiorgan damage [1, 5, 8, 9]. Dermal histopathology has identified epidermal necrosis, subcutaneous haemorrhage, fibrinoid small arteriole necrosis, mixed inflammatory infiltrate and occasional thrombosis [1, 5, 8].

The aetiology is unknown although multiple possibilities have been proposed, including ingestion of bacteria-associated shiga toxin [1, 2, 4, 5, 7, 8, 10] However, studies have failed to consistently identify the presence of shiga toxin, or bacteria or viruses within tissues from affected cases [1, 4, 5]. Electron microscopy with immunostaining has also failed to identify immune-complex deposition in affected cases [4, 5, 8]. Despite this, several risk factors for development of the condition have been identified, including being from the hound or gundog Kennel Club groups, and certain individual breeds such as English springer spaniel, flat-coated retriever, Hungarian vizsla and whippet, and being female and neutered [10]. The majority of diagnoses are made between November and May, and case distribution is strongly linked to woodland habitats [11]. In addition, multiple cases within the same household have been reported [5].

The following report describes three dogs diagnosed with CRGV in the Republic of Ireland. As cases of CRGV have not been described to date within this country, this report is intended to raise awareness of this condition as a differential diagnosis for dogs presenting with skin lesions and/or azotaemia. In addition, this report is the first to describe TMA in the cerebrum of a dog presenting with seizures as a result of CRGV.

## Case presentation

Signalment, demographics and prior history of all three cases is presented in Table 1. Full clinicopathological data and medications administered are included as supplementary data in Additional file 1: Tables S1-S4*.*

**Table 1:** Signalment, demographics and relevant clinical history of three dogs with cutaneous and renal glomerular vasculopathy presented to University College Dublin Veterinary Hospital between 2014 and 2018

	Case One	Case Two	Case Three
Age (years)	4	4	0.5
Breed	Hungarian vizsla	golden retriever	Hungarian vizsla
Sex	F(E)	F (N)	M (E)
Weight (kg)	31.0	32.2	20.4
Body condition score (/5)	3	3	2.5
Location	Tara, County Meath	Ranelagh, South Dublin	Navan, County Meath
Walking environment	Woodland, County Meath	Woodland, Southwest Dublin	Equine yard exercise
Other household pets	One cat	One dog	
Vaccine status	Up to date	Up to date	Up to date
Antiparasitic therapy	Up to date	Up to date	Up to date
Travel history	Mainland Europe, 10 months prior to presentation	None	None
Previous medical history	Snake bite during European travel 10 months prior to presentation	None	None
Presenting clinical signs at primary veterinarian	Ventral skin lesion, lethargy, inappetence, vomiting, pyrexia	Left hindlimb lameness, ventral skin lesion, lethargy, inappetence, vomiting, pyrexia	Distal thoracic limb skin lesions, lethargy, anorexia, vomiting, pyrexia
Clinicopathological abnormalities recorded at primary veterinary practice		Neutrophilia, monocytosis, thrombocytopenia, increased urea concentration, hyperbilirubinaemia, increase in hepatic enzyme activities, proteinuria, haematuria	Thrombocytopenia, azotaemia, hypoalbuminaemia

### Case one

**Fig. 1:**
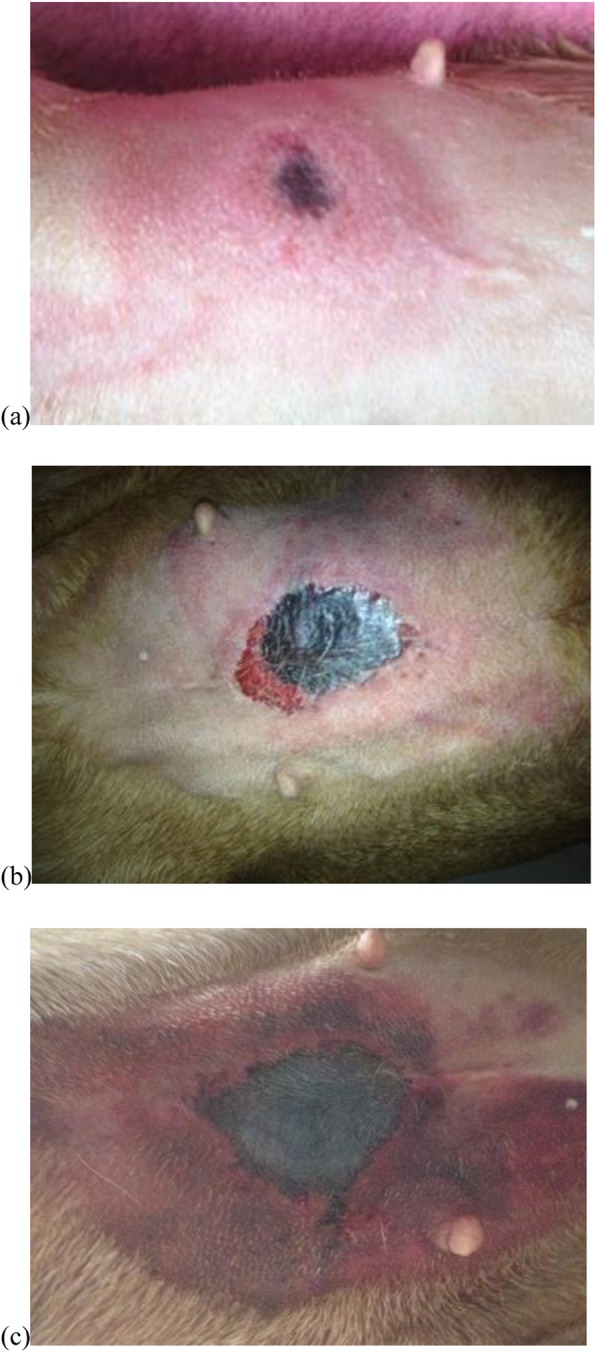
Images of a dog with CRGV (Case 1) illustrating how a dark-red necrotic, cutaneous lesion in the umbilical region expanded in size over the course of approximately 48 h (**a**-**c**). Credit: Gregory Cameron MVB, AllPets Drogheda

A 4-year-old intact female Hungarian viszla was presented to UCDVH in June 2014 with a black, necrotic, cutaneous lesion, of approximately 2 cm in diameter, involving the umbilical region of the ventral abdomen (Fig. 1). The dog was dull, moderately dehydrated and icteric on presentation. There were extensive ecchymoses on the ventral abdomen and ventrolateral aspect of the thorax bilaterally.

Haematology, biochemistry, urinalysis and coagulation testing identified thrombocytopenia (19 (reference interval 150–500) × 10^9^/L), azotaemia (creatinine 283 (20–120) umol/L urea 40.6 (3.6–8.6) mmol/L, panhypoproteinaemia (total protein 41.8 (54–71) g/L, hyperbilirubinaemia (258 (0.9–10) umol/L, increased creatine kinase (CK) (1590 (0–122) U/L and hepatic enzyme activities (alkaline phosphatase (ALP) 4200 (0–82) U/L, alanine transaminase (ALT) 1087 U/L; RI 0–36, glutamate dehydrogenase (GLDH) 370 (0–16) U/L, aspartate aminotransferase (AST) 749 (0–37) U/L), prolonged prothrombin time (17.2 (7–14) s), glucosuria (3+) and proteinuria (3+). tick-borne disease (*Babesia spp., Anaplasma spp., Ehrlichia* spp. and *Hepatozoon canis*) polymerase chain reaction (PCR) (IDEXX Laboratories, Wetherby, United Kingdom) and *Leptospira* microscopic agglutination test (MAT) (Agri-Food and Biosciences Institute Veterinary Laboratory, Belfast, Northern Ireland) were negative. Thoracic and abdominal imaging identified microcardia and echogenic material in the urinary bladder consistent with haemorrhage.

Standard management for AKI was undertaken (fluid therapy, antiemetics, gastroprotectants and diuretic), in addition to antimicrobial therapy (amoxicillin-clavulanate and doxycycline) given the possibility of leptospirosis, whilst awaiting test results. However the dog continued to deteriorate, with worsening anaemia (haematocrit 0.16 (0.37–0.55) L/L), azotaemia (creatinine 691 umol/L, urea 57 mmol/L, and hyperbilirubinaemia (425 umol/L), and euthanasia was performed at the owner’s request.

Pertinent gross post mortem findings included ecchymoses within the renal capsule and bladder wall in addition to congested and oedematous pulmonary parenchyma. Histopathological examination revealed microthrombosis and necrosis of renal glomerular tufts and, multifocally, of tubular epithelia. Transmural haemorrhage was confirmed in the bladder and occasional microthrombi were observed in the lung. Randomly distributed focal hepatocyte necrosis and loss was also noted. At the time, CRGV was not well recognised and retrospective evaluation of post-mortem data was required to confirm a diagnosis.

### Case two

**Fig. 2:**
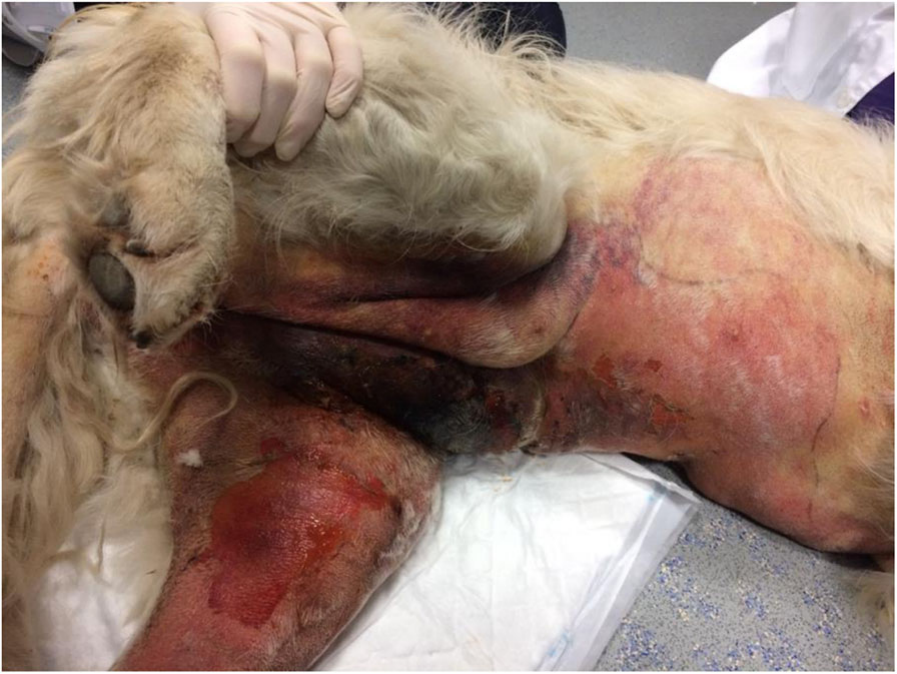
Images of a dog with CRGV (Case 2) illustrating an extensive, erythematous necrotic lesion involving the skin of the caudoventral abdomen and medial aspect of left hindlimb

A 4-year-old neutered female golden retriever was presented to UCDVH in February 2017 with a large, necrotic, cutaneous lesion involving the ventral abdomen and left hindlimb, extending from the caudal ventral abdomen to the mid-aspect of the ventrum (Fig. 2). Pitting oedema was present on the left hindlimb. The dog was dull, poorly ambulatory and icteric. Multiple, widespread petechiae were identified.

Haematology, biochemistry, urinalysis and coagulation testing identified non-regenerative anaemia (haematocrit 0.34 L/L), thrombocytopenia (19 × 10^9^/L), leucocytosis (white blood cell count 25.01 (6–17) × 10^9^/L), increased urea concentration (15.3 mmol/L), panhypoproteinaemia (total protein 41.2 g/L), hyperbilirubinaemia (237.2 umol/L), increased CK (3041 U/L) and hepatic enzyme activities (ALP 915 U/L, ALT 141 U/L, AST 598 U/L), glucosuria (2+), proteinuria (2+) and granular casts in the urine. Assessment for canine pancreatic lipase concentration (cPLI) (SNAP™) (IDEXX Laboratories) was abnormal (quantitative value (IDEXX Laboratories, Wetherby, United Kingdom) 234 (< 200) ug/L in the equivocal range).

The anaemia, renal parameters and hyperbilirubinaemia (haematocrit 0.29 L/L, creatinine 181 umol/L, urea 22.7 mmol/L, bilirubin 336.9 umol/L) worsened over the first 24 h. Standard therapy for AKI was administered (fluid therapy, antiemetics, gastroprotectants and diuretics), however the urine output declined (to 0.8 mL/kg/hour) and creatinine increased (305 umol/L) despite this therapy. The owners opted to euthanase the dog on the third day of therapy.

Post mortem examination revealed fibrinoid necrosis and microthrombosis within renal glomeruli, hepatic sinusoids and the stomach and small intestinal walls with attendant haemorrhage, necrosis and inflammation. There was marked dermal oedema and inflammation with necrosis of the overlying epidermis. Multifocal necrosis, haemorrhage and inflammation were noted in the subcutis where fibrinoid necrosis of arterioles was a feature.

### Case three

**Fig. 3:**
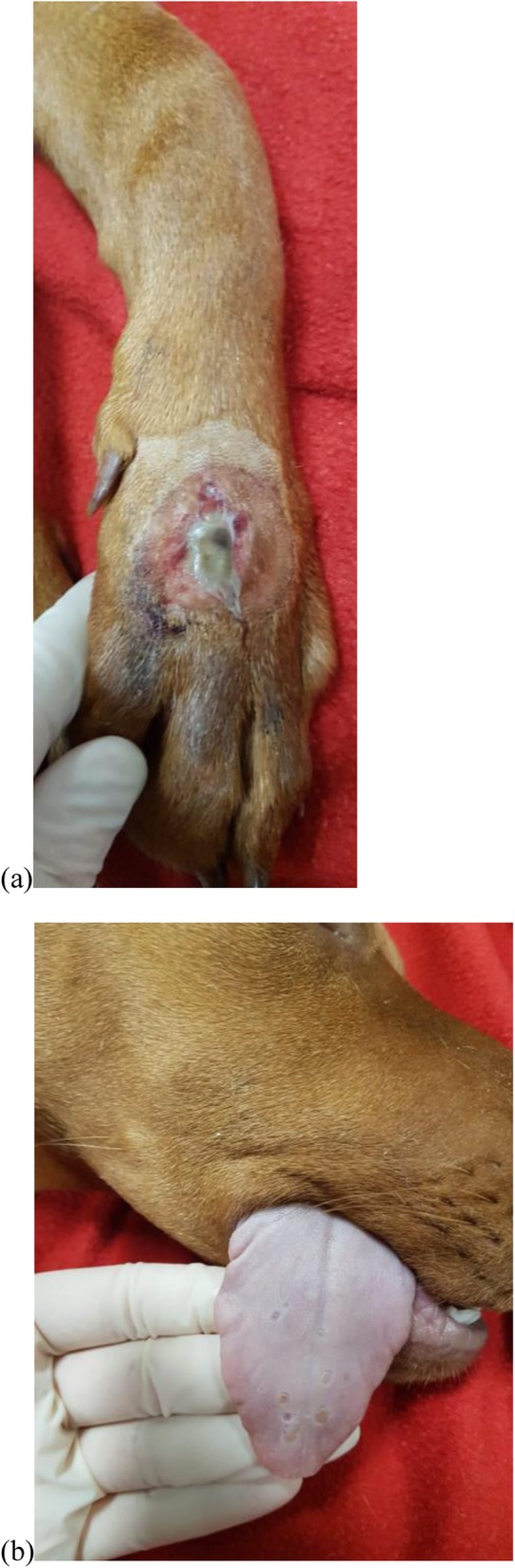
Images of a dog with CRGV (Case 3) illustrating: **a** well-demarcated, approximately 2 cm diameter ulcerated and exudative skin lesion on the dorsolateral aspect of the left carpus; and **b** multifocal ulceration of the dorsum of the tongue

A 6-month-old entire male Hungarian viszla was presented to UCDVH in October 2018 with a well-demarcated, ulcerative and exudative skin lesion on the dorsolateral aspect of the left carpus (Fig. 3). A smaller lesion of similar appearance was also noted at the palmar aspect of the left thoracic paw, and there were multiple small ulcers on the tongue.

Haematology, biochemistry, urinalysis and coagulation testing identified azotaemia (creatinine 179 umol/L, urea 31.1 mmol/L), hyperphosphataemia (3.87 (0.8–1.8) mmol/L), proteinuria (3+) and granular casts in the urine. SNAP™ cPLi was abnormal (quantitative value > 2000 μg/L; consistent with pancreatitis). Leptospira SNAP™ test, 4DX SNAP™ test (IDEXX Laboratories), Angiodetect™ (IDEXX Laboratories) and modified Baermann were negative. Abdominal ultrasonography identified moderately thickened and diffusely hyperechoic renal cortices with a hyperechoic rim at the cortico-medullary junction, bilaterally.

The dog was managed with antimicrobial (amoxicillin-clavulanate) and intravenous fluid therapy. Seizure episodes developed on the second day of hospitalisation, with three occurring in the space of a 24 h period. There was no change in mentation after the development of seizure episodes, however the dog was very dull throughout the hospitalisation time. Unfortunately, a neurological examination was not performed. Over 3 days of therapy, the azotaemia markedly worsened (creatinine 412 umol/L, urea 60.6 mmol/lL) and thrombocytopenia developed (manual platelet count 75 × 10^9^/L). Due to the progression of clinical and clinicopathological abnormalities, the owners elected for euthanasia.

**Fig. 4:**
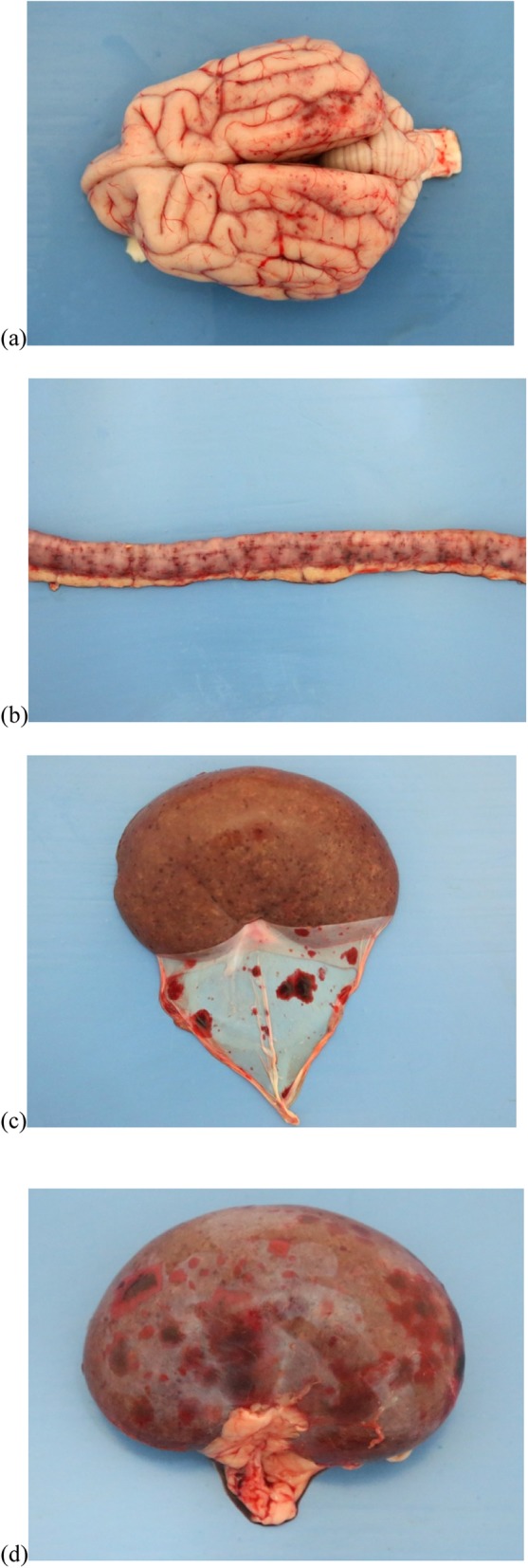
Images following postmortem examination of a dog with CRGV (Case 3) illustrating petechiation/ecchymoses of: **a** dorsal leptomeninges; **b** small intestinal serosa; and **c** and **d** renal capsule and cortex

Pertinent gross post mortem findings included petechiae and ecchymoses of the gastric and intestinal mucosa, renal cortices and leptomeninges (Fig. 4*)*. Histopathological examination revealed arteriolar fibrinoid necrosis and microthrombosis within renal glomeruli, in the myocardium, lung and pancreas, transmurally within the small intestine, within the dermis and subcutis, and focally within the cerebrum. There were varying degrees of attendant haemorrhage, necrosis and inflammation.

## Discussion and conclusions

The three dogs in this report represent the first cases of CRGV to be described in the Republic of Ireland. Two of the three dogs were Hungarian vizslas, which have previously been reported to be at increased risk for the development of the disease [10]. Similar to previous reports of association between development of CRGV and walking in woodland habitats [11], two of the three cases were known to have walked in woodland in the days prior to the development of clinical signs. One dog was housed with another dog, although unlike previous reports where multiple dogs from the same household were affected [5], the remaining dog did not develop the condition.

All three dogs presented with a variety of cutaneous lesions, evidence of AKI (either at presentation or developing rapidly after the onset of the cutaneous lesions) and thrombocytopenia. Two of the three cases were hyperbilirubinaemic. These clinicopathological findings are similar to those described in previous CRGV cases [1–5]. In addition, cPLi SNAP™, performed in two of the three dogs, was abnormal in both (within the equivocal range, and consistent with pancreatitis, in cases two and three, respectively on quantification). This is similar to a previous report, in which all CRGV cases in which cPLi testing was performed had abnormal results. It is possible that the abnormal cPLi concentration in CRGV cases could result from reduced glomerular filtration rate, as pancreatic lipase has previously been demonstrated to increase in some dogs with experimentally-induced AKI [12]. However, the pancreatic microthrombosis as observed in Case Three (the dog with the highest quantitative result) could also be considered as a possible mechanism for increased pancreatic lipase activity.

With regards to possible infectious aetiologies, all three dogs were tested for leptospirosis and vector-borne diseases, all with negative results. This is similar to previous reports in which there has been no consistent identification of an infectious aetiology [1, 4, 5]. Similarly, bacterial culture of the skin lesions of two of the three cases did not identify any growth of *E. coli* to support the possibility of infection with shiga-like toxin-producing *E. coli* species. Unfortunately, faecal culture was not performed in any of the cases.

All three dogs were examined post-mortem and had lesions consistent with a diagnosis of CRGV (characteristic renal TMA). Thrombosis was also identified within the intestinal vasculature of Cases Two and Three, and hepatic vasculature of Case Two. To the author’s knowledge, the intestinal and hepatic abnormalities have not previously been reported. The intestinal pathology could contribute to the gastrointestinal signs (inappetence, vomiting) that precede development of azotaemia in some CRGV cases.

One dog developed seizure episodes shortly after admission. This has been described in previous CRGV cases [4, 9], with histopathological analysis of neural tissue revealing mild non-specific changes [4] and multifocal neuronal degeneration and necrosis with haemorrhage and oedema [9]. In case three, histopathology of the cerebrum identified a focal small region of microthrombosis with associated necrosis, microhaemorrhage and inflammation of adjacent grey matter, consistent with TMA. This is the first publication to document TMA in the cerebral vessels of a dog with CRGV, and suggests a possible aetiology for the seizure episodes documented.

The condition has been noted to occur in clusters with regard to temporal and spatial factors when groups of CRGV cases have been examined [11], Two clusters occurred in the region of the New Forest, UK in February – March 2013 and April 2015–2017 and one cluster in Manchester, UK in February – April 2014. Although these clusters may have been artefactually created due to awareness and vigilance in the area at these times, there remains a possibility that a factor within the environment, be that infectious, toxic or otherwise, is contributing to the development of CRGV. Interestingly, two of the three cases presented in this report resided within a seven-mile radius of each other although presenting 4 years apart.

In conclusion, this case series is the first to describe dogs with CRGV presenting within the Republic of Ireland. It is hoped that this raises awareness of the presence of the condition within this country, and aids practitioners in the recognition of CRGV. Earlier recognition will aid therapy and may help to improve prognosis.

## Supplementary information

**Additional file 1 Table S1.** Clinicopathological data throughout 5 days of hospitalisation in a dog with cutaneous and renal glomerular vasculopathy (case one), pCO_2_: partial pressure of carbon dioxide, pO_2_: partial pressure of oxygen, HCO_3_^-act^: bicarbonate, Hct: haematocrit, Hgb: haemoglobin, RBC: red blood cells, MCHC: mean corpuscular haemoglobin concentration, MCV: mean corpuscular volume, MCH: mean corpuscular haemoglobin, ALP: alkaline phosphatase, CK: creatine kinase, GGT: gamma glutamyl transferase, ALT: alanine aminotransferase, GLDH: glutamate dehydrogenase, AST: aspartate aminotransferase, WBC: white blood cells, PT: prothrombin time, aPTT: activated partial thromboplastin time, MAT: microscopic agglutination test, PCR: polymerase chain reaction **Table S2.** Clinicopathological data throughout 3 days of hospitalisation in a dog with cutaneous and renal glomerular vasculopathy (case two), pCO_2_: partial pressure of carbon dioxide, pO_2_: partial pressure of oxygen, HCO_3_^-act^: bicarbonate, Hct: haematocrit, Hgb: haemoglobin, RBC: red blood cells, MCHC: mean corpuscular haemoglobin concentration, MCV: mean corpuscular volume, MCH: mean corpuscular haemoglobin, ALP: alkaline phosphatase, CK: creatine kinase, GGT: gamma glutamyl transferase, ALT: alanine aminotransferase, GLDH: glutamate dehydrogenase, AST: aspartate aminotransferase, WBC: white blood cells, PT: prothrombin time, aPTT: activated partial thromboplastin time, FDP: fibrinogen degradation products, cPLi: canine pancreatic lipase **Table S3.** Clinicopathological data throughout 3 days of hospitalisation in a dog with cutaneous and renal glomerular vasculopathy (case three), pCO_2_: partial pressure of carbon dioxide, pO_2_: partial pressure of oxygen, HCO_3_^-act^: bicarbonate, Hct: haematocrit, Hgb: haemoglobin, RBC: red blood cells, MCHC: mean corpuscular haemoglobin concentration, MCV: mean corpuscular volume, MCH: mean corpuscular haemoglobin, ALP: alkaline phosphatase, CK: creatine kinase, GGT: gamma glutamyl transferase, ALT: alanine aminotransferase, GLDH: glutamate dehydrogenase, AST: aspartate aminotransferase, WBC: white blood cells, PT: prothrombin time, aPTT: activated partial thromboplastin time, ACTH: adrenocorticotropic hormone, cPLi: canine pancreatic lipase **Table S4.** In hospital management of three dogs with cutaneous and renal glomerular vasculopathy.

## Data Availability

Not applicable.

## Abbreviations

AKI: Acute kidney injury
ALP: Alkaline phosphatase
ALT: Alanine aminotransferase
AST: Aspartate aminotransferase
CK: Creatine kinase
cPLi: Canine pancreatic lipase
CRGV: Cutaneous and renal cutaneous vasculopathy
GLDH: Glutamate dehydrogenase
MAT: Microscopic agglutination test
PCR: Polymerase chain reaction
TMA: Thrombotic microangiopathy
UCDVH: University College Dublin Veterinary Hospital
UK: United Kingdom
