# Supplementary material for: Canine cutaneous and renal glomerular vasculopathy in the Republic of Ireland: a description of three cases
Source: Ir Vet J. 2019 Nov 16;72:13. doi: 10.1186/s13620-019-0151-7 (PMC6858974; doi:10.1186/s13620-019-0151-7)
Supplement: Supplementary file 1 — Additional file 1 Table S1. Clinicopathological data throughout 5 days of hospitalisation in a dog with cutaneous and renal glomerular vasculopathy (case one), pCO2: partial pressure of carbon dioxide, pO2: partial pressure of oxygen, HCO3-act: bicarbonate, Hct: haematocrit, Hgb: haemoglobin, RBC: red blood cells, MCHC: mean corpuscular haemoglobin concentration, MCV: mean corpuscular volume, MCH: mean corpuscular haemoglobin, ALP: alkaline phosphatase, CK: creatine kinase, GGT: gamma glutamyl transferase, ALT: alanine aminotransferase, GLDH: glutamate dehydrogenase, AST: aspartate aminotransferase, WBC: white blood cells, PT: prothrombin time, aPTT: activated partial thromboplastin time, MAT: microscopic agglutination test, PCR: polymerase chain reaction Table S2. Clinicopathological data throughout 3 days of hospitalisation in a dog with cutaneous and renal glomerular vasculopathy (case two), pCO2: partial pressure of carbon dioxide, pO2: partial pressure of oxygen, HCO3-act: bicarbonate, Hct: haematocrit, Hgb: haemoglobin, RBC: red blood cells, MCHC: mean corpuscular haemoglobin concentration, MCV: mean corpuscular volume, MCH: mean corpuscular haemoglobin, ALP: alkaline phosphatase, CK: creatine kinase, GGT: gamma glutamyl transferase, ALT: alanine aminotransferase, GLDH: glutamate dehydrogenase, AST: aspartate aminotransferase, WBC: white blood cells, PT: prothrombin time, aPTT: activated partial thromboplastin time, FDP: fibrinogen degradation products, cPLi: canine pancreatic lipase Table S3. Clinicopathological data throughout 3 days of hospitalisation in a dog with cutaneous and renal glomerular vasculopathy (case three), pCO2: partial pressure of carbon dioxide, pO2: partial pressure of oxygen, HCO3-act: bicarbonate, Hct: haematocrit, Hgb: haemoglobin, RBC: red blood cells, MCHC: mean corpuscular haemoglobin concentration, MCV: mean corpuscular volume, MCH: mean corpuscular haemoglobin, ALP: alkaline phosphatase, CK: creatine kinase, GGT: gamma glutamyl tr [file 13620_2019_151_MOESM1_ESM.docx]

**Additional file 1**

| ***Blood gas (venous)*** | **Day One** | **Day Two** | **Day Five** | **Reference intervals** |
| --- | --- | --- | --- | --- |
| *pH* | 7.424 | 7.37 | **7.27** | 7.35 – 7.44 |
| *pCO_2_* | **3.45** | **4.42** | **4.06** | 4.47 – 5.48kPa |
| *pO_2_* | **7.72** | **10.52** | **9** | 6.37 – 7.49kPa |
| *HCO_3_^- act^* | **16.6** | **18.8** | **15.9** | 20.8 – 24.2mmol/l |
| *Base excess* | **-7.8** | **-9.8** | **-11** | 0 +/- 4 |
| ***Haematology*** |  |  |  |  |
| *Hct* | 0.46 | **0.28** | **0.16** | 0.37 – 0.55l/L |
| *Hgb* | 166 | **103** | **57** | 120 – 180g/L |
| *RBC* | 6.4 | **3.93** | **2.2** | 5.5 – 8.5 x 10^12^/l |
| *MCHC* | **363** | **372** | **364** | 310 – 362g/L |
| *MCV* | 71.6 | 70.3 | 71.4 | 60 – 77 fL |
| *MCH* | **26** | **26.1** | **26** | 19.5 – 25pg |
| *Reticulocytes* | 21.5 | 13.5 | 14.2 | 0 – 60 x 10^9^/l |
| *Platelets* | **19** | **21** | **95** | 150 – 500 x 10^9^/l |
| *Leucocytes* | 12.81 | 10.08 | 16.14 | 6 – 17 x 10^9^/l |
| *Neutrophils* | 8.97 | 7.86 | **14.2** | 3 – 11.5 x 10^9^/l |
| *Band neutrophils* | 0.13 | - | 0.32 |  |
| *Lymphocytes* | **0.51** | **0.2** | **0.32** | 1 – 3.6 x 10^9^/l |
| *Monocytes* | **3.07** | **2.02** | 1.29 | 0 – 1.35 x 10^9^/l) |
| *Eosinophils* | - | - | - | 0 – 1.47 x 10^9^/l |
| *Smear report* | Hypersegmentation of neutrophils. Marked thrombocytopenia confirmed on smear. | Hypersegmentation of neutrophils. Marked thrombocytopenia with rare macroplatelet. | Mild anisocytosis with occasional dacrocyte, microcyte and 1+ macrocytes. Non-regenerative anaemia at this time. Manual platelet count 150 x 10^9^/l |  |
| ***Biochemistry*** |  |  |  |  |
| *Comment* | Icteric |  | Icteric |  |
| *Total protein* | **41.8** | **35.2** | **35.1** | 54 – 71g/l |
| *Globulin* | **17.5** | **13.4** | **12.9** | 28 – 42g/l |
| *Calcium* | **2.14** | **2.12** | **2.03** | 2.3 – 3mmol/l |
| *Creatinine* | **283** | **425** | **691** | 20 – 120umol/l |
| *Amylase* | **1633** | **3064** | **15140** | 400 – 1300U/L |
| *Cholesterol* | **6.79** | 4.44 | **2.69** | 3.2 – 6.5mmol/l |
| *ALP* | **4200** | **3037** | **1455** | 0 – 82U/L |
| *Total bilirubin* | **258** | **350.8** | **425** | 0.9 – 10umol/l |
| *Phosphorous* | **2.69** | **3.15** | **3.81** | 0.8 – 1.8mmol/l |
| *CK* | **1590** | **693** | **443** | 0 – 122U/L |
| *Sodium* | 138 | **133.7** | 140.7 | 137 – 151mmol/l |
| *Chloride* | **100.7** | **100.5** | 111.3 | 105 – 117mmol/l |
| *Anion Gap* | 18.7 | 17.7 | 22.69 | 11 – 26mmol/l |
| *Albumin* | **24.3** | **21.8** | **22.2** | 25 – 38g/l |
| *A:G ratio* | **1.3** | **1.6** | **1.7** | 0.59 – 1.11 |
| *Urea* | **40.6** | **47** | **57** | 3.6 – 8.6mmol/l |
| *Lipase* | 99 | **360** | **1397** | 0 – 130U/L |
| *Glucose* | **9.4** | **10** | **6.7** | 3 – 6.5mmol/l |
| *Triglycerides* | 0.56 | 0.52 | 0.82 | 0.11 – 1.69mmol/l |
| *GGT* | 0 | 13 | 0 | 0 – 16U/L |
| *ALT* | **1087** | **717** | **328** | 0 – 36U/L |
| *GLDH* | **370** | **187** | **44** | 0 – 16U/L |
| *AST* | **749** | **303** | **248** | 0 – 37U/L |
| *Potassium* | 3.9 | 4.11 | 5.49 | 3.7 – 5.8mmol/l |
| ***Urinalysis*** |  |  |  |  |
| *Colour* |  | Dark yellow | Amber |  |
| *Odour* |  | Normal | Normal |  |
| *Turbidity* |  | Sl. cloudy | Sl. cloudy |  |
| *Specific gravity* | **1.018** | **1.020** | **1.010** |  |
| *Nitrite* |  | - | - |  |
| *pH* |  | 8.0 | 6.5 |  |
| *Epithelial* |  | Few – 1+ | 1+ |  |
| *RBC* |  | **2-3+** | **3+** |  |
| *WBC* |  | Few | Few |  |
| *Protein* |  | **3+** | **1+** |  |
| *Glucose* |  | **3+** | **2+** |  |
| *Ketones* |  | - | - |  |
| *Urobilinogen* |  | - | - |  |
| *Bilirubin* |  | **3+** | **2+** |  |
| *Blood* |  | **3+** | **4+** |  |
| *Casts* |  | - | **Few granular** |  |
| *Crystals* |  | - | - |  |
| *Bacteria* |  | - | - |  |
| ***Coagulation testing*** |  |  |  |  |
| *PT* | **17.2** | 13.5 | 12.9 | 7 – 14s |
| *aPTT* | 20.4 | 19.7 | 19.1 | 12 – 25s |
| ***Other*** |  |  |  |  |
| *Leptospirosis MAT* | Negative for Bratislava, Canicola, Hardjo-bovis and Icterohaemorrhagica |  |  |  |
| *Babesia PCR* | Negative |  |  |  |
| *Anaplasma PCR* | Negative |  |  |  |
| *Ehrlichia spp. PCR* | Negative |  |  |  |
| *Hepatozoon canis PCR* | Negative |  |  |  |

**Table S1:** Clinicopathological data throughout five days of hospitalisation in a dog with cutaneous and renal glomerular vasculopathy (case one), pCO_2_: partial pressure of carbon dioxide, pO_2_: partial pressure of oxygen, HCO_3_^-act^: bicarbonate, Hct: haematocrit, Hgb: haemoglobin, RBC: red blood cells, MCHC: mean corpuscular haemoglobin concentration, MCV: mean corpuscular volume, MCH: mean corpuscular haemoglobin, ALP: alkaline phosphatase, CK: creatine kinase, GGT: gamma glutamyl transferase, ALT: alanine aminotransferase, GLDH: glutamate dehydrogenase, AST: aspartate aminotransferase, WBC: white blood cells, PT: prothrombin time, aPTT: activated partial thromboplastin time, MAT: microscopic agglutination test, PCR: polymerase chain reaction

| ***Blood gas (venous)*** | **Day One** | **Day Two** | **Day Three** | **Reference intervals** |
| --- | --- | --- | --- | --- |
| *pH* | **7.53** | **7.5** | 7.43 | 7.35 – 7.44 |
| *pCO_2_* | 4.94 | 5.2 | 4.5 | 4.47 – 5.48kPa |
| *pO_2_* | **4.04** | **4.6** | **2.25** | 6.37 – 7.49kPa |
| *HCO_3_^- act^* | **30.4** | **30.5** | 22.3 | 20.8 – 24.2mmol/l |
| *Base excess* | **7.7** | **7.5** | -1.8 | 0+/-4 |
| ***Haematology*** |  |  |  |  |
| *Hct* | **0.34** | **0.29** | **0.19** | 0.37 – 0.55l/L |
| *Hgb* | 122 | **106** | **70** | 120 – 180g/L |
| *RBC* | **4.94** | **4.26** | **2.76** | 5.5 – 8.5 x 10^12^/l |
| *MCHC* | 355 | **370** | **373** | 310 – 362g/L |
| *MCV* | 69.2 | 67.2 | 68 | 60 – 77 fL |
| *MCH* | 24.6 | 24.9 | **25.4** | 19.5 – 25pg |
| *Reticulocytes* | 24.4 | 24.5 | 76.9 | 0 – 60 x 10^9^/l |
| *Platelets* | **19** | **28** | **66** | 150 – 500 x 10^9^/l |
| *Leucocytes* | **35.23** | **37.78** | **47.71** | 6 – 17 x 10^9^/l |
| *Neutrophils* | **25.01** | **29.09** | **30.06** | 3 – 11.5 x 10^9^/l |
| *Band neutrophils* | 2.47 | 3.4 | 10.97 |  |
| *Lymphocytes* | **0.7** | 1.51 | 2.86 | 1 – 3.6 x 10^9^/l |
| *Monocytes* | **6.69** | **3.4** | **3.82** | 0 – 1.35 x 10^9^/l) |
| *Eosinophils* | 0.35 | 0.38 | - | 0 – 1.47 x 10^9^/l |
| *Smear report* | Some neutrophils highly segmented. Manual platelet count one per x100 field, macroplatelets only | Mild toxic changes. Anisocytosis. Thrombocytopenia – rare macroplatelet observed on smear. Agglutination test – negative | Four nucleated red blood cells per 100 white blood cells. Manual platelet count approximately as machine count – many macroplatelets. Anisocytosis with macrocytes, polychromatic red blood cells, schistocytes and red blood cell fragments. Rare very large mononuclear cell |  |
| ***Biochemistry*** |  |  |  |  |
| *Comment* | Icteric | Icteric and haemolysed | Icteric and haemolysed |  |
| *Total protein* | **41.2** | **39.7** | **31.1** | 54 – 71g/l |
| *Globulin* | **20.4** | **18.5** | **13.5** | 28 – 42g/l |
| *Calcium* | **2.26** | 2.36 | **2.22** | 2.3 – 3mmol/l |
| *Creatinine* | 109 | **181** | **305** | 20 – 120umol/l |
| *Amylase* | 959 | 1289 |  | 400 – 1300U/L |
| *Cholesterol* | 4.34 | 4.17 |  | 3.2 – 6.5mmol/l |
| *ALP* | **915** | **640** |  | 0 – 82U/L |
| *Total bilirubin* | **237.2** | **336.9** | **390.3** | 0.9 – 10umol/l |
| *Phosphorous* | 1.57 | 1.7 | 1.76 | 0.8 – 1.8mmol/l |
| *CK* | **3041** | **1879** |  | 0 – 122U/L |
| *Sodium* | 141.1 | 137.3 | 139.2 | 137 – 151mmol/l |
| *Chloride* | **101.6** | **96.4** | 108.2 | 105 – 117mmol/l |
| *Anion Gap* | 14.61 | 15.89 |  | 11 – 26mmol/l |
| *Albumin* | **20.8** | **21.2** | **17.6** | 25 – 38g/l |
| *A:G ratio* | 1.019 | **1.14** | **1.3** | 0.59 – 1.11 |
| *Urea* | **15.3** | **22.7** | **35.1** | 3.6 – 8.6mmol/l |
| *Lipase* | 40 | 67 |  | 0 – 130U/L |
| *Glucose* | 6.9 | 5.4 |  | 3 – 6.5mmol/l |
| *Triglycerides* | 0.58 | 0.85 |  | 0.11 – 1.69mmol/l |
| *GGT* | 0 | 0 |  | 0 – 16U/L |
| *ALT* | 141 | 116 |  | 0 – 36U/L |
| *GLDH* | 15 | 10 |  | 0 – 16U/L |
| *AST* | 598 | 675 |  | 0 – 37U/L |
| *Potassium* | 4.11 | 3.09 | 5.5 | 3.7 – 5.8mmol/l |
| ***Urinalysis*** |  |  |  |  |
| *Colour* | Dark yellow |  |  |  |
| *Odour* | Normal |  |  |  |
| *Turbidity* | Clear |  |  |  |
| *Specific gravity* | **1.011** |  |  |  |
| *Nitrite* | - |  |  |  |
| *pH* | 8.0 |  |  |  |
| *Epithelial* | 1-2+ |  |  |  |
| *RBC* | - |  |  |  |
| *WBC* | 1-2+ |  |  |  |
| *Protein* | **2+** |  |  |  |
| *Glucose* | **2+** |  |  |  |
| *Ketones* | - |  |  |  |
| *Urobilinogen* | - |  |  |  |
| *Bilirubin* | **3+** |  |  |  |
| *Blood* | **3+** |  |  |  |
| *Casts* | **Few granular/hyaline** |  |  |  |
| *Crystals* | - |  |  |  |
| *Bacteria* | - |  |  |  |
| ***Coagulation testing*** |  |  |  |  |
| *PT* | 10.5 |  |  | 7 – 14s |
| *aPTT* | 18.9 |  |  | 12 – 25s |
| *FDP* |  | 5 - 20 |  | 0 - 20 |
| *D-dimers* |  | 2.06 |  | 1 - 4 |
| *Fibrinogen* |  | 0.2 |  | 0 – 0.5 |
| ***Other*** |  |  |  |  |
| *cPLi SNAP^TM^* | **Abnormal** |  |  |  |
| *Quantitative cPLi* | **234** |  |  | <200ug/l |
| *Leptospirosis SNAP^TM^* | Negative |  |  |  |
| *Angiodetect^TM^* | Negative |  |  |  |
| *Modified Baermann* | Negative |  |  |  |

**Table S2:** Clinicopathological data throughout three days of hospitalisation in a dog with cutaneous and renal glomerular vasculopathy (case two), pCO_2_: partial pressure of carbon dioxide, pO_2_: partial pressure of oxygen, HCO_3_^-act^: bicarbonate, Hct: haematocrit, Hgb: haemoglobin, RBC: red blood cells, MCHC: mean corpuscular haemoglobin concentration, MCV: mean corpuscular volume, MCH: mean corpuscular haemoglobin, ALP: alkaline phosphatase, CK: creatine kinase, GGT: gamma glutamyl transferase, ALT: alanine aminotransferase, GLDH: glutamate dehydrogenase, AST: aspartate aminotransferase, WBC: white blood cells, PT: prothrombin time, aPTT: activated partial thromboplastin time, FDP: fibrinogen degradation products, cPLi: canine pancreatic lipase

| ***Haematology*** | **Day One** | **Day Three** | **Reference intervals** |
| --- | --- | --- | --- |
| *Hct* | 0.4 | 0.4 | 0.37 – 0.55l/L |
| *Hgb* | 137 | 137 | 120 – 180g/L |
| *RBC* | 5.78 | 5.79 | 5.5 – 8.5 x 10^12^/l |
| *MCHC* | 346 | 342 | 310 – 362g/L |
| *MCV* | 68.3 | 69.1 | 60 – 77 fL |
| *MCH* | 23.7 | 23.6 | 19.5 – 25pg |
| *Reticulocytes* | 30.8 | **71.1** | 0 – 60 x 10^9^/l |
| *Platelets* | **66** | **24** | 150 – 500 x 10^9^/l |
| *Leucocytes* | 15.96 | **27.87** | 6 – 17 x 10^9^/l |
| *Neutrophils* | **11.97** | **20.07** | 3 – 11.5 x 10^9^/l |
| *Band neutrophils* | - | **0.56** |  |
| *Lymphocytes* | 1.92 | 2.51 | 1 – 3.6 x 10^9^/l |
| *Monocytes* | **1.92** | **4.74** | 0 – 1.35 x 10^9^/l) |
| *Eosinophils* | 0.16 | - | 0 – 1.47 x 10^9^/l |
| *Smear report* | Normal morphology of red and white blood cells. 1+ macroplatelets. Manual platelet count 150 x 10^9^/L | Leucocytosis with mature neutrophilia, rare band neutrophil and monocytosis. Mild anisocytosis. 1+ macroplatelets. Manual platelet count 75 x 10^9^/L |  |
| ***Biochemistry*** |  |  |  |
| *Comment* | - | - |  |
| *Total protein* | 54.1 | **48.7** | 54 – 71g/l |
| *Globulin* | **26.3** | **25.3** | 28 – 42g/l |
| *Calcium* | 2.96 | 2.66 | 2.3 – 3mmol/l |
| *Creatinine* | **179** | **412** | 20 – 120umol/l |
| *Amylase* | 1188 |  | 400 – 1300U/L |
| *Cholesterol* | **8.16** | **6.82** | 3.2 – 6.5mmol/l |
| *ALP* | **434** | **203** | 0 – 82U/L |
| *Total bilirubin* | 3.1 | 0 | 0.9 – 10umol/l |
| *Phosphorous* | **3.87** | **4.8** | 0.8 – 1.8mmol/l |
| *CK* | **486** | **8179** | 0 – 122U/L |
| *Sodium* | 144.3 | 144.5 | 137 – 151mmol/l |
| *Chloride* | **104.5** | **102.8** | 105 – 117mmol/l |
| *Anion Gap* | 19.74 | 22.56 | 11 – 26mmol/l |
| *Albumin* | 27.8 | **23.4** | 25 – 38g/l |
| *A:G ratio* | 1.06 | 0.92 | 0.59 – 1.11 |
| *Urea* | **31.1** | **60.6** | 3.6 – 8.6mmol/l |
| *Lipase* | 33 | **299** | 0 – 130U/L |
| *Glucose* | **7.23** | 5.58 | 3 – 6.5mmol/l |
| *Triglycerides* | 0.51 | 1.05 | 0.11 – 1.69mmol/l |
| *GGT* | 0 | 0 | 0 – 16U/L |
| *ALT* | **89** | **147** | 0 – 36U/L |
| *GLDH* | **23.3** | 0 | 0 – 16U/L |
| *AST* | **84** | **982** | 0 – 37U/L |
| *Potassium* | 3.84 | 4.96 | 3.7 – 5.8mmol/l |
| ***Urinalysis*** |  |  |  |
| *Colour* | Dark yellow | Red |  |
| *Odour* | Normal | Normal |  |
| *Turbidity* | Cloudy | Cloudy |  |
| *Specific gravity* | > 1.050 | **1.018** |  |
| *Nitrite* | - | - |  |
| *pH* | 9 | 8 |  |
| *Epithelial* | 1-2+ | 1+ |  |
| *RBC* | 1+ | **4+** |  |
| *WBC* | 1+ | 1+ |  |
| *Protein* | **3+** | **3+** |  |
| *Glucose* | - | **1-2+** |  |
| *Ketones* | - | - |  |
| *Urobilinogen* | - | - |  |
| *Bilirubin* | - | - |  |
| *Blood* | **4+** | 4+ |  |
| *Casts* | **Few granular, waxy and cellular** | - |  |
| *Crystals* | - | - |  |
| *Bacteria* | Sparse | - |  |
| *Urine protein:creatinine ratio* | **3.2** |  | <0.5 |
| ***Coagulation testing*** |  |  |  |
| *PT* |  | 9.4 | 7 – 14s |
| *aPTT* |  | 21.8 | 12 – 25s |
| *D-dimers* |  | 0.2 | 0 – 0.5mg/l |
| ***Other*** |  |  |  |
| *ACTH stimulation test* | Results precluded hypoadrenocorticism |  |  |
| *cPLi SNAP^TM^* |  | Abnormal |  |
| *Quantitative cPLi (RI <200ug/l)* |  | > 2000 | <200ug/l |
| *Leptospirosis SNAP^TM^* | Negative |  |  |
| *Dirofilaria immitis antigen* | Negative |  |  |
| *Anaplasma phagocytophilum antibody titre* | Negative |  |  |
| *Anaplasma platys titre* | Negative |  |  |
| *Borrelia burgdorferi titre* | Negative |  |  |
| *Ehrlichia canis titre* | Negative |  |  |
| *Ehrlichia ewingii titre* | Negative |  |  |
| *Angiodetect* | Negative |  |  |
| *Modified Baermann* | Negative |  |  |

**Table S3:** Clinicopathological data throughout three days of hospitalisation in a dog with cutaneous and renal glomerular vasculopathy (case three), pCO_2_: partial pressure of carbon dioxide, pO_2_: partial pressure of oxygen, HCO_3_^-act^: bicarbonate, Hct: haematocrit, Hgb: haemoglobin, RBC: red blood cells, MCHC: mean corpuscular haemoglobin concentration, MCV: mean corpuscular volume, MCH: mean corpuscular haemoglobin, ALP: alkaline phosphatase, CK: creatine kinase, GGT: gamma glutamyl transferase, ALT: alanine aminotransferase, GLDH: glutamate dehydrogenase, AST: aspartate aminotransferase, WBC: white blood cells, PT: prothrombin time, aPTT: activated partial thromboplastin time, ACTH: adrenocorticotropic hormone, cPLi: canine pancreatic lipase

|  | **Case One** | **Case Two** | **Case Three** |
| --- | --- | --- | --- |
| *Intravenous fluid therapy* | Compound sodium lactate with potassium supplementation | Compound sodium lactate and 0.9% sodium chloride with potassium supplementation | Compound sodium lactate |
| *Blood products* | Fresh frozen plasma |  |  |
| *Antimicrobials* | Amoxicillin-clavulanate, doxycycline | Amoxicillin-clavulanate, metronidazole, marbofloxacin | Amoxicillin-clavulanate |
| *Antiemetics/gastroprotectants* | Metoclopramide, omeprazole, sucralfate | Maropitant, omeprazole | Maropitant, omeprazole |
| *Analgesia* | Methadone | Fentanyl |  |
| *Diuretics* | Mannitol, furosemide | Mannitol, furosemide |  |
| *Other* | Ursodeoxycholic acid, dexamethasone |  | Diazepam, levetiracetam |

**Table S4**: In hospital management of three dogs with cutaneous and renal glomerular vasculopathy
